# Exogenous spatial attention is functional in paralytic strabismics

**DOI:** 10.3389/fnins.2025.1650468

**Published:** 2025-09-22

**Authors:** Kevin T. Willeford, Robert M. McPeek

**Affiliations:** ^1^Department of Optometric Sciences, NOVA Southeastern University College of Optometry, Fort Lauderdale, FL, United States; ^2^Department of Biological and Vision Sciences, SUNY State College of Optometry, New York, NY, United States

**Keywords:** exogenous attention, paralytic strabismus, premotor theory, overt attention, covert attention

## Abstract

**Introduction:**

The absence of exogenous cueing effects in patients with paralytic strabismus has been cited as evidence for an obligate linkage between movement execution and attentional allocation.

**Methods:**

The present study challenges this interpretation through two experiments that measured the impact of visual cues, intended to facilitate or inhibit shifts of spatial attention, on the latencies of target-directed responses. By including a no-cue control condition, we assessed both the presence and direction of cue-induced attentional modulation.

**Results:**

Our results show that all patients’ patterns of saccadic (Experiment I) and keypress (Experiment II) response latencies showed classic signatures of cue-target interactions: movements initiated in response to targets spatially proximate to cues were initiated fastest whereas movements initiated toward targets spatially distal from cues were initiated slowest. This trend held true when grouping latencies by cue-saccade offset to account for small errors in saccade landing position in Experiment I. Though the patients’ data exhibited small degrees of idiosyncrasy, most patients’ mean normalized latencies also fell within the range of latencies observed in non-strabismic participants.

**Discussion:**

These findings support the notion that premotor structures may program attentional shifts in an effector-independent manner and motivate future investigations to explore how, not if, exogenous spatial attention is deployed in paralytic strabismics.

## 1 Introduction

Exogenous spatial attention allows us to quickly orient ourselves toward salient objects (Chica et al., 2011; Macaluso and Doricchi, 2013). Its presence is captured with cue-target paradigms in which a preceding cue in the same or opposite hemi-field (“side”) as a target either facilitates (speeds up) or inhibits (slows down) the latencies of subsequent goal-directed movements (Posner and Cohen, 1984). The neurophysiological basis for the observed latency modulations is believed to involve dynamic competition within neural priority maps, in which visual cues transiently increase the activity of neurons representing specific spatial locations, biasing the outcome of competitive interactions which determine movement initiation (Bisley and Mirpour, 2019; Ptak, 2012; Sprague and Serences, 2013). Thus, in cue-target paradigms, movements planned to spatial locations in which a visual stimulus was recently presented are executed faster because the visual cue-evoked activity, fed forward to visuomotor or motor neurons representing that specific spatial location (McPeek and Keller, 2002; Schall, 1995), gives these movement-initiating neurons a “head start” when instructions for the subsequent goal-directed movement arrive (Fecteau and Munoz, 2006; White and Munoz, 2017). Conversely, movements planned to spatial locations distant from the visual cues take longer to initiate as neural competition within the priority map potentially delays or alters movement initiation (Dorris et al., 2007; White et al., 2012). It is these spatiotemporal interactions between precedent visual cues and subsequent motor plans on the priority map that create the facilitatory and inhibitory latency modulations used as signatures of exogenous spatial attention in psychophysical paradigms. The cues, which initially summon neural activity to a specific area of the priority map, speed up or slow down an observer’s response more-so than if no cue was present (Khan et al., 2010).

Patients with paralytic strabismus have historically presented a compelling means to examine the interaction between precedent visual cue-evoked activity and motor programming. This is because a longstanding hypothesis, the premotor theory, posits an obligatory linkage between motor planning and deployment of spatial attention. A key assumption unifying prior investigations of visual attention in paralytic strabismics was that the observed movement restrictions in this population, deficits in movement execution, reflected gaze-specific absences of motor programming. This presumed absence of motor programming was then purported to explain the apparent absence of spatial attention in this clinical population. Support for this interpretation comes primarily from covert exogenous cueing paradigms in which individuals are surmised to plan, but not execute, eye movements toward cued or uncued locations. For example, Rafal et al. (1988) showed that patients with progressive supranuclear palsy (PSP) exhibited smaller, albeit present, cueing effects when cues and targets were presented in the vertical meridian in which eye movements were restricted (Rafal et al., 1988). Similarly, Smith et al. (2004) found that a patient with congenital ophthalmoplegia, unable to execute eye movements in any direction, failed to display latency modulations when cues were presented in the same vs. opposite hemi-field as target locations (Smith et al., 2004). Finally, a similar absence of cueing effects was documented by Gabay et al. (2010) in three patients with Duane Retraction Syndrome (DRS), a congenital sixth nerve palsy associated with unilateral abduction deficits (Gabay et al., 2010). All three of these investigations concluded that a lack of motor planning (evidenced as movement restrictions), and thus deployment of spatial attention, could explain the absence of cueing effects.

Several methodological limitations within these prior investigations limit the strength of this conclusion. First, because covert cueing paradigms require individuals to maintain fixation throughout the experiment, latency modulations at distinct target locations serve only as indirect proxies by which to detect deployment of spatial attention. In other words, covert cueing paradigms cannot directly reveal where exogenous attention is allocated or capture changes if deployment shifts to other locations (McFadden et al., 2002). Second, because each investigation presented stimuli at only two spatial locations, it is possible that exogenous attention was deployed to, but not detected at, other locations in space. Third, because none of the three prior investigations included no-cue control conditions, it is not possible to know whether the absence of cueing effects, observed as equivalent latencies in both the same- and opposite-side cue conditions, were due to absences of facilitation, inhibition, or both. Together, these limitations leave the presence of facilitatory and/or inhibitory signatures of exogenous attention unexplored across the full extent of spatial locations in paralytic strabismics.

More broadly, it remains unclear whether the philosophical underpinnings of the premotor theory provide an appropriate framework within which to interpret these findings. Emerging evidence is challenging the assumption that the capability for ocular motor movement execution is necessary for the allocation of exogenous spatial attention (Cooper and McPeek, 2021; Song and McPeek, 2015; Song et al., 2011). First, movement planning and movement execution are not obligatorily linked together (Klein, 2020). The absence of movement execution (i.e., presence of movement restrictions) in paralytic strabismics does not necessarily imply that motor planning itself is altered or absent. For example, the causative lesion in DRS, a unilateral absence of the abducens nucleus, occurs downstream of premotor structures in the brainstem. This, in conjunction with functional internuclear neurons on this affected side (Yüksel et al., 2010), endow the unaffected “sound” eye with the ability to execute normal gain saccades into both the unaffected and affected fields (Yüksel et al., 2005). This suggests that premotor circuits may continue to generate accurate movement plans even if execution is impaired downstream (Yüksel et al., 2008). This was not assessed in any of the prior experiments because eye movements were not measured in either the sound or affected eye. Second, because many patients with paralytic strabismus adapt by executing gaze shifts with head-saccades (Barry et al., 2019; Khan et al., 2009; Smith et al., 2004), both attentional allocation and motor programming likely remain functional, as goal directed movements are still planned and executed, albeit primarily with the head (Khan et al., 2009), across the entire visual field. This is supported by a recent investigation by Hanning et al. (2019) which found that exogenous cues modulated attention both within and outside of the oculomotor range of participants. Third, and relatedly, there is evidence that the neural structures responsible for allocating spatial attention toward impending movement goals operate in an effector-independent fashion (Cooper and McPeek, 2021). Thus, if the brain uses such effector-independent priority maps to plan and execute movements (Song and McPeek, 2015; Song et al., 2011), then an abnormality in a single peripheral effector should not, by itself, disrupt attentional allocation toward an entire region of visual space. Fourth, it must be considered that deployment of exogenous attention may not require motor programming at all [i.e., disembodied cognition (Klein, 2020)].

These theoretical and methodological limitations motivated us to develop an exogenous cueing paradigm capable of detecting attentional signatures, across multiple spatial locations, in patients with paralytic strabismus. Our design also allowed us to test whether cue-evoked shifts of attention depend on the effector used to report them. By comparing patterns of saccadic and keypress response latencies, we examined three possibilities: (1) if exogenous attention depends on functional eye movement execution, cueing effects should be absent in the affected hemi-field in both response types, (2) if exogenous attention is allocated in an effector-specific way, cueing effects should be absent only in saccades directed toward the affected hemi-field and (3) if exogenous attention is indeed effector-independent, both modalities should show intact cueing effects in all hemi-fields despite impaired eye movement execution.

## 2 Materials and methods

### 2.1 Patients and participants

Five strabismic patients with congenital (4 total, 1 female, aged 29–56) or acquired (1 male, aged 40) sixth nerve palsies and ten non-strabismic participants (5 females, aged 22–30) were recruited from the clinical and student populations at the SUNY State College of Optometry. Each was required to possess best-corrected visual acuity of at least 20/30 in each eye. A range of motility restrictions, described in Table 1, was present in the patient population. Four of the five total patients took part in each of the two planned experiments. All patients and participants took part in an informed consent discussion prior to data collection and written informed consent was obtained from all patients and participants. The experimental protocol was approved by the Institutional Review Board at the SUNY State College of Optometry and adhered to the tenets of the Declaration of Helsinki.

**TABLE 1:** Demographics and diagnoses of patient population.

Patient	Experiments	Demographics, diagnosis and motility defect
A	I, II	29 year-old male with DRS Type I; impaired abduction in the right eye since birth.
B	I, II	29 year-old male with DRS Type I; impaired abduction in the left eye since birth.
C	I, II	45 year-old female with DRS Type I; impaired abduction in the left eye since birth.
D	I	40 year-old male with acquired sixth nerve palsy; impaired abduction in the left eye. He underwent radiosurgery 2 years following the onset of an acute left esotropia secondary to a benign meningioma at the level of the left abducens nucleus.
E	II	56 year-old male with bilateral DRS Type I; impaired abduction in both eyes since birth.

All patients had restricted motility in at least one direction of gaze, with four of the five patients possessing these restrictions since birth (A–C and E) and one with an acquired and persistent motility defect following treatment of a benign meningioma (D).

### 2.2 Procedure

#### 2.2.1 Exogenous cueing paradigms

We implemented two exogenous cueing paradigms that required individuals to indicate the location of a visual target via either saccadic eye movements (Experiment I) or keypresses (Experiment II). In each paradigm, targets were presented on either the same (“valid,” evoking facilitation and shorter response latencies) or opposite (“invalid,” evoking inhibition and prolonged response latencies) hemi-field as preceding visual cues, presented at multiple spatial locations to evoke varying degrees of latency modulation (Boehnke and Munoz, 2008; Khan et al., 2010). The two paradigms differed in the instructed response (i.e., saccade or keypress). Cue-evoked attentional modulations occur when using either response modality; however, each uses a different stimulus-response mapping. Responses executed with saccades utilize a “direct” mapping in which the motor effector is moved to the same area of visual space as target presentation, whereas responses executed with keypresses require an “indirect” mapping which symbolically links areas of visual space to manual movements of the finger (Khatoon et al., 2002).

#### 2.2.2 Trial structure

**FIGURE 1:**
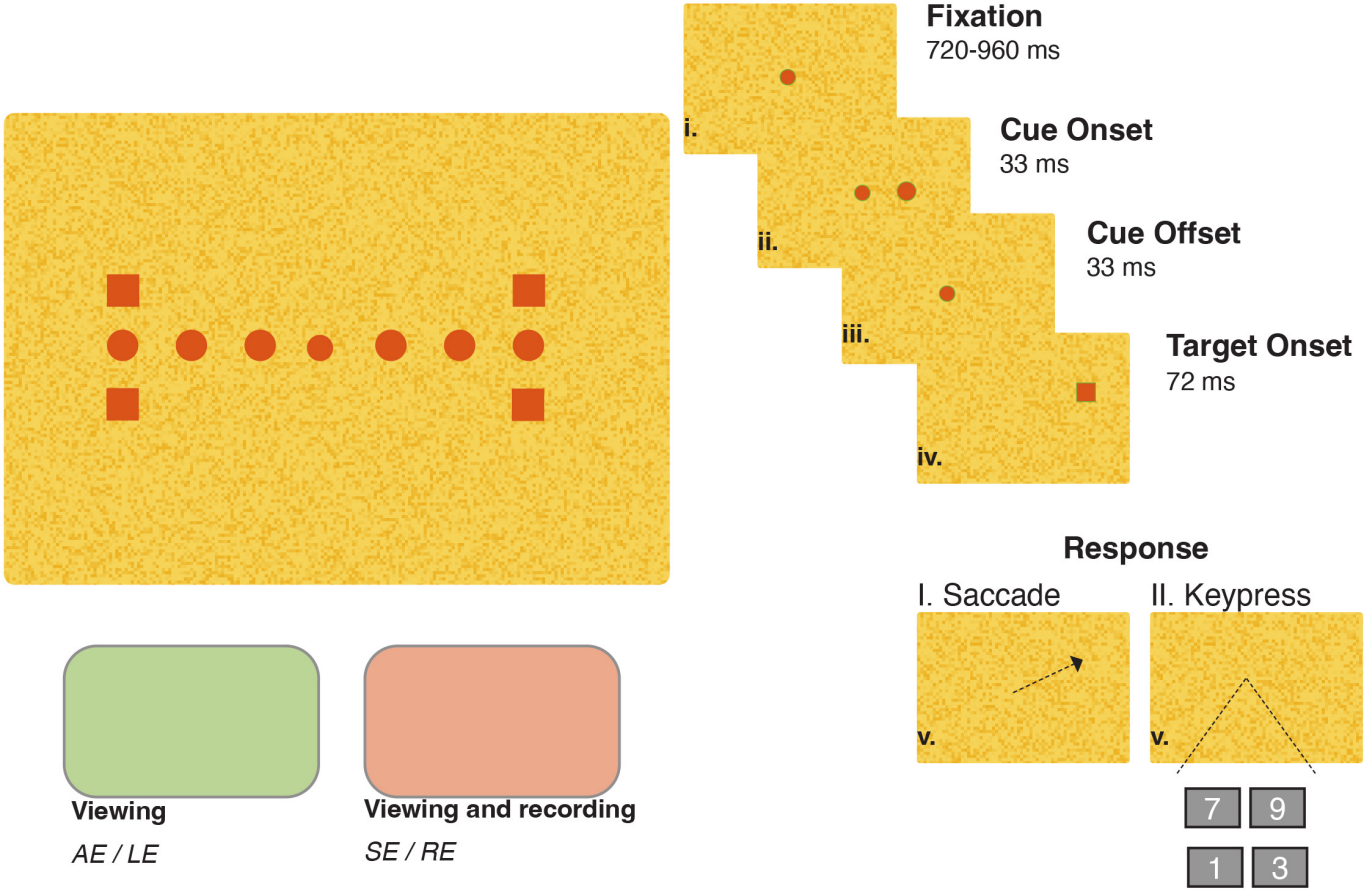
Trial structure, stimulus and set-up for a right-eye same side trial. One fixation point (central circle), four target locations (squares, 10° horizontal eccentricity and 1.25 ° above or below the horizontal midline), and six cue positions (circles, 5°, 7.5°, or 10° horizontal eccentricity) were used in both viewing conditions. The right side of the panel illustrates the stimulus sequence in a same-side trial. The stimulus color was dependent on the viewing eye condition: red for the sound eye (SE) or right eye (RE) and green for the affected eye (AE) or left eye (LE) in patients and participants, respectively.

We utilized a trial sequence equivalent to that of prior spatially extended exogenous cueing experiments (Khan et al., 2010). Figure 1 shows a schematic of the trial structure and stimulus display. All trials in each experiment began with monocular fixation of a 0.30° diameter central circle. Fixation, always recorded from the sound eye, was required to be maintained within a small window surrounding the fixation point for between 1.5 and 2 s in both paradigms. This window size was adjusted on an individual basis to allow for the presence of small fixation inaccuracies and heterophorias, manifest when participants and patients viewed with their left (participants) or affected (patients) eye, a set-up detailed in see section “2.2.3 Stimuli and set-up.” The diameter of the window, centered around the fixation point, was between 3 and 5° for all participants and patients except for Patient C for whom we set the diameter to 6° when viewing with her sound right eye. A brief (33 ms) visual cue then appeared at one of six locations: 5°, 7.5°, or 10° degrees to the right or left of fixation. In both experiments, premature fixation losses or saccades initiated prior to target appearance resulted in abortion of the current trial with a subsequent repetition. Next, following a short 66 ms cue-target onset asynchrony (CTOA), a square target (1° wide) appeared simultaneously with fixation offset at one of four locations 1.25° above or below the 10°cue locations. The short CTOA was chosen because previous neurophysiological (Fecteau and Munoz, 2005) and behavioral (Khan et al., 2010) investigations have shown that this interval produces facilitation of goal-directed responses spatially coincident with precedent visual cues. The four-alternative forced choice trial structure was implemented to minimize the proportion of premature anticipatory responses. Then, after onset, the target was extinguished after a duration of 72 ms to prevent individuals from receiving visual feedback regarding their goal-directed accuracy, a potential stimulus for short-term attentional (McFadden et al., 2002) or motor adaptation (Noto and Robinson, 2001). Last, patients and participants indicated the location of the visual target via a saccadic eye movement to the remembered location of the target (Experiment I) or via a symbolic keypress onto one of four possible keys (1, 3, 7, or 9 for the lower left, lower right, upper left, and upper right, respectively) representing the four target locations (Experiment II). A seventh no-cue condition, in which accurate fixation triggered onset of only the target, was interleaved in both experiments to assess response latencies without the influence of a precedent visual cue. The memory-based nature of the saccade task in Experiment I prevented strict online error control from being implemented; however, as detailed in our subsequent analyses, the diversity of saccadic landing points elicited allowed us to investigate the influence of saccadic accuracy on latency modulations. Conversely, because it was always possible to judge a keypress as correct or incorrect, only correct trials were accepted during Experiment II. Trials in which participants or patients pressed the incorrect key prompted a “re-do” of the trial.

#### 2.2.3 Stimuli and set-up

Stimulus presentation was performed monocularly to segregate potential differences in attentional modulation related to patients viewing with their sound- vs. affected-eyes (Craighero et al., 2001; Gabay et al., 2010). This was achieved via dichoptic viewing in which red and green dichroic filters (Bernell Corporation) segregated visual input to either the right- or left-eye. Each patient’s filter configuration was dependent on the type of paralytic restriction for two reasons. First, because the limited motility of the affected eye potentially prevents the full magnitude of the motor command from being observed, the full extent of a motor command can only be inferred from recording movements of the sound eye (Optican et al., 1985). Second, because the infrared light emitted by the Eyelink 1000 (SR Research, Mississuaga, Canada), used for eye tracking, was blocked by the green filter, the red filter was always placed in front of patients’ fully mobile unaffected sound eye. This was the right eye for patients with left sixth nerve palsies (B, C, and D) and the left eye for Patient A with a right sixth nerve palsy. Patient E, with bilateral restrictions, wore the red filter on his right eye and partook only in Experiment II. The right eye of all non-strabismic participants was used for recording. Thus, all patients and participants, with the exception of Patient A, had their right-eyes recorded, viewed red stimuli with their right eye and viewed green stimuli with their left eye. Saccades and keypresses were detected online, using Eyelink and Psychopy respectively, with latencies computed as the time elapsed between target appearance and initiation of either a saccadic or keypress response. Eye movements were recorded during both experiments to monitor fixation and to detect anticipatory and/or target-directed saccades. Online saccade detection was performed using the EYELINK parser’s velocity and acceleration thresholds of 35°/s and 9500°/s^2^, respectively. In Experiment I, the first detected saccade in each trial was chosen for analysis. For this experiment, there were infrequent occurrences in which abnormally long-duration trajectories were marked as saccades; thus, saccades with durations greater than 100 ms (3 and 4% of participants’ and patients’ data, respectively) were excluded from further analysis.

During all trials, patients and participants were seated 114 cm in front of a 32” Display + + LCD monitor with a refresh rate of 120 Hz (Cambridge Research Systems Ltd., Kent, UK). The size and spatial location of the fixation point, cues, and saccade targets were identical across all conditions. The luminance and chromaticity coordinates of the stimuli within each viewing condition were as follows: red viewed through red filter: [5.18 cd/m^2^, CIE x, y = 0.677, 0.360], red viewed through green filter: [1.20 cd/m^2^, CIE x, y = 0.227, 0.600], green viewed through green filter: [5.20 cd/m^2^, CIE x, y = 0.172, 0.710], green viewed through red filter: [0.86 cd/m^2^, CIE x, y = 0.617, 0.314]. A textured noisy background (1.05 and 0.90 cd/m^2^ through the red and green filters, respectively) ensured that each stimulus was rendered imperceptible by the non-viewing eye.

Each individual, with the exception of Patients D (two sessions) and E (one session), completed a total of three sessions. Experiment I was completed in two sessions: patients and participants viewed with either their sound- vs. affected- or right- vs. left-eyes, respectively, during the first [sound and right] and second [affected and left] sessions. The number of cue- and target-locations resulted in a total of 28 possible trial types repeated up to 35 times each for a total of 980 possible trials per viewing condition. Data for Experiment II were collected under both viewing conditions during the third session. A possible total of 476 trials were collected per viewing condition with each of the 28 possible trial types being repeated up to 17 times.

#### 2.2.4 Data analysis

The primary objective of our analyses was to determine whether visual cues modulated patients’ saccadic or keypress latencies. To quantify this, we calculated normalized response latencies by subtracting the mean latencies recorded in the no-cue control conditions from those recorded in cue-present trials, separately for each target location, viewing eye, and response modality combination. This normalization allowed us to isolate the impact of each cue location on response latency.

For each response modality, we grouped normalized latencies by target location (right or left hemi-field, collapsed across vertical target positions) and by viewing eye (right or left). This resulted in the creation of up to 24 normalized latency distributions per patient, each reflective of how each of the six cue locations influenced responses to targets in either hemi-field under each monocular viewing condition. To reduce fatigue in three patients (C–E), only four cue locations (the 5° and 10° eccentricities in both hemi-fields) were tested, yielding 16 normalized distributions instead. We interpreted negative normalized latencies (i.e., faster responses relative to the no-cue condition) as evidence of facilitation and positive normalized latencies (i.e., slower responses) as evidence of inhibition in movement initiation.

We used Kruskal-Wallis tests, separately for Experiment I and Experiment II, to test for differences in normalized latencies across cue locations in each viewing eye x target location condition in our non-strabismic group. These analyses, described subsequently, confirmed our experimental design evoked shifts of exogenous attentional in our normal population. Then, to evaluate if and how the normalized latencies of our patient population deviated from this typical pattern, we computed z-scores comparing each patient’s mean normalized latency, for each cue location within each condition, to the corresponding distribution of data found in our normal population. Once computed, these scores indicate how much each patient’s data points deviate from the normative mean, with positive z-scores reflecting slower reaction times above the mean and negative z-scores reflecting faster reaction times below it.

## 3 Results

### 3.1 Experiment I

The mean no-cue saccadic latencies, expressed as 95% confidence intervals, ranged between 251 to 279 ms (right eye viewing right target), 243 to 275 ms (right eye viewing left target), 230 to 253 ms (left eye viewing right target) and 230 to 257 ms (left eye viewing left target) in non-strabismic individuals and were not significantly different when compared across these conditions [H(3) = 2.01, *p* = 0.57 via Kruskall-Wallis test]. Patients’ mean no-cue saccadic latencies, reported in the same order, were as follows: Patient A (223, 212, 239 and 244 ms), Patient B (307, 317, 301 and 314 ms), Patient C (323, 271, 313 and 269 ms), and Patient D (221, 253, 258 and 273 ms). The differential no-cue saccade latency trends in Patient C (shorter response latencies when executing saccades into the affected left hemi-field) and Patient D (shortest responses for targets in the right hemi-field), while outside the cue-dependency foci of the current investigation, do suggest that hemi-field and/or viewing-eye dependent differences in general alertness may be additional areas for future exploration in paralytic strabismics.

**FIGURE 2:**
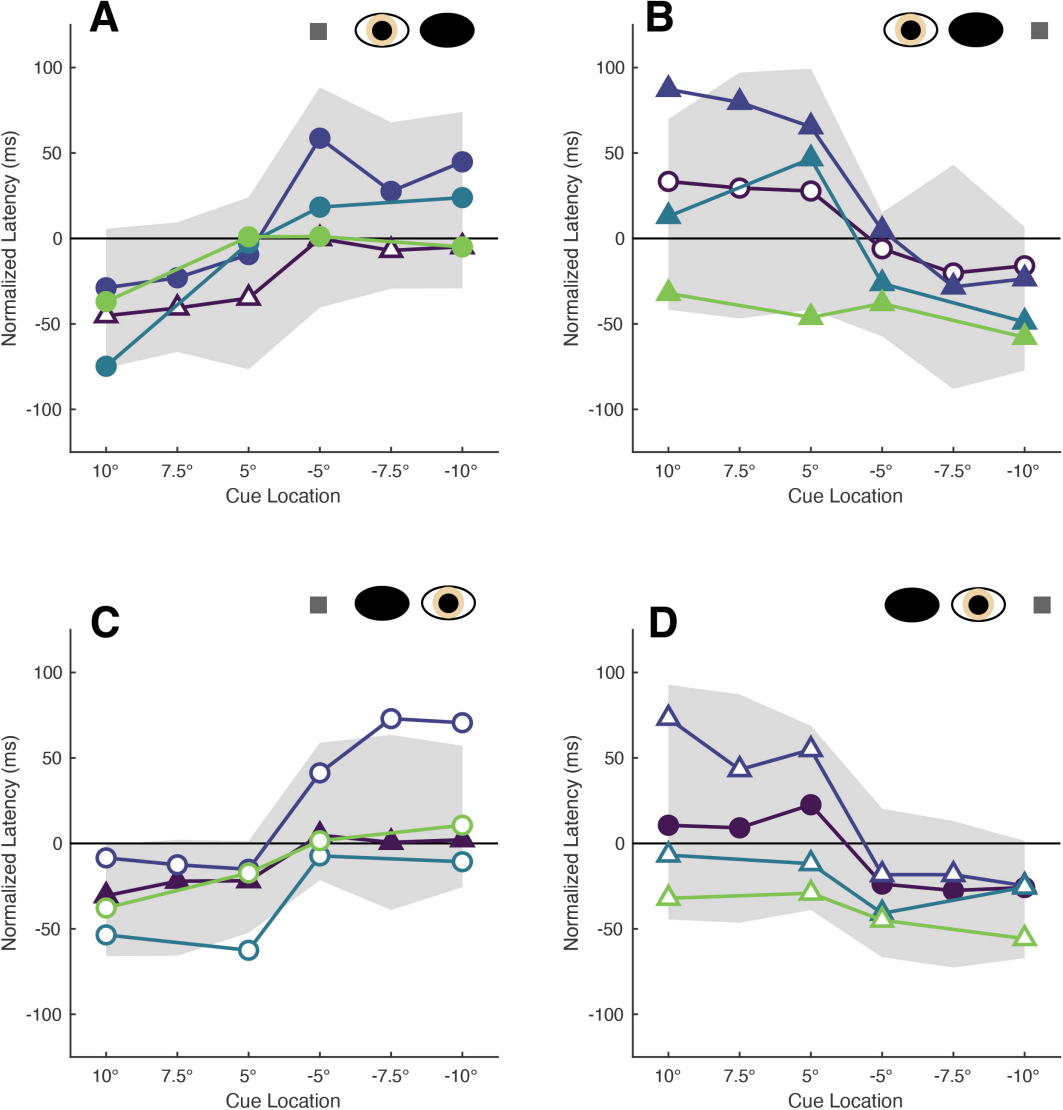
The latencies of patients’ target-directed saccades are modulated by cue position. Each panel shows the mean normalized latencies of all patients (Patient A: purple, Patient B: dark blue, Patient C: blue, Patient D: green) for each viewing eye [right eye in panel **(A,B)**, left eye in panel **(C,D)**] and target location [rightward targets in panel **(A,C)**, leftward targets in panel **(B,D)**] condition. Panels are oriented such that one is viewing the patient: rightward target locations are shown to the left, and indicated with positive numbers on the abscissa, and vice versa. The shading and shape of datapoints differentiate the sound vs. affected eyes (filled vs. unfilled) and sound vs. affected hemi-fields (circles vs. triangles). The gray shaded region represents the 95% confidence interval obtained from sampling the mean latencies of the non-strabismic participants.

Normalization of the cue-present latencies showed that saccadic latencies in all patients and participants were modulated by cue position: cues closest to target locations reduced latencies relative to the no-cue control condition, whereas cues distant from target locations tended to increase latencies (Figure 2). Kruskall-Wallis tests conducted to examine differences in normalized latency across each of the six cue positions in our non-strabismic population revealed statistically significant differences across all viewing eye x target location conditions [H(5) = 32.91, 29.94, 38.59 and 29.25 for right eyes viewing right and left targets and left eyes viewing right and left targets with all *p* < 0.001, respectively]. *Post hoc* comparisons using the Tukey-Kramer method confirmed that same- and opposite-side cues differentially modulated saccadic latencies with few exceptions. This finding is enumerated in Table 2 which shows that significant comparisons (*p* < 0.05) tended to “cluster in the corners” of each condition’s comparison matrix. This clustering occurs because cues presented in the same hemi-field as targets evoked significantly faster response latencies than cues presented in the opposite hemi-field.

**TABLE 2:** Results of *post-hoc* testing comparing non-strabismic participants’ mean normalized saccadic latencies across cue locations within each viewing eye and target location condition.

Condition	*Post-hoc* comparisons						
RE/RHF		10°	7.5°	5°	−5°	−7.5°	−10°
	10°				*	*	*
	7.5°				*	*	*
	5°				*	*	*
	−5°	*	*	*			
	−7.5°	*	*	*			
	−10°	*	*	*			
RE/LHF		10°	7.5°	5°	−5°	−7.5°	−10°
	10°						*
	7.5°					*	*
	5°					*	*
	−5°						
	−7.5°		*	*			
	−10°	*	*	*			
LE/RHF		10°	7.5°	5°	−5°	−7.5°	−10°
	10°				*	*	*
	7.5°				*	*	*
	5°				*	*	*
	−5°	*	*	*			
	−7.5°	*	*	*			
	−10°	*	*	*			
LE/LHF		10°	7.5°	5°	−5°	−7.5°	−10°
	10°					*	*
	7.5°					*	*
	5°					*	*
	−5°						
	−7.5°	*	*	*			
	−10°	*	*	*			

Asterisks indicate significant comparisons (*p* < 0.05) within each viewing eye (RE, right eye; LE, left eye) x target location (RHF, right hemi-field; LHF, left hem-field) condition.

The normalized latencies of each patient generally fell no more than two standard deviations away from the respective mean normalized latency computed from non-strabismic individuals. Moreover, as displayed in Figure 2 and enumerated in Table 3, the small number of deviations that did occur were in the direction predicted by functional exogenous spatial attention: same- vs. opposite hemi-field cues sometimes evoked much faster or slower response latencies than those of the normative group. The pattern of cue-evoked latency modulations was generally consistent regardless of whether patients viewed with their sound or affected eye, or into their sound or affected hemi-field; thus, no clear dependence on viewing eye or hemi-field was observed. However, closer inspection reveals that facilitation was observed more frequently than inhibition. For several patients, invalid opposite hemi-field cues, those most distant from target locations, failed to increase response latencies above that of the no-cue condition. This is evidenced in Patient A’s saccades directed to targets in his affected hemi-field: cues presented in the left hemi-field did not inhibit movement initiation. A similar pattern was present when Patient C viewed with her affected eye and for Patient D in most conditions. Thus, though all patients displayed both inhibition and facilitation in at least one condition, the specific pattern of latency modulation varied individually. This variability also extended to the comparisons between each patient and the non-strabismic control group: the relative normality of each patient’s degrees of inhibition and facilitation were idiosyncratic. It is important to note that, independent of the specific patterns of facilitation and inhibition across cue positions, cues most proximal to visual targets always reduced saccadic latencies more so than the most distal cue for all patients and non-strabismic participants.

**TABLE 3:** Z-scores for individual patients’ normalized saccadic latencies across viewing eye and target location conditions.

Patient	Condition	Right cues			Left cues		
		10°	7.5°	5°	−5°	−7.5°	−10°
A	RE/RHF	−0.50	−0.65	−0.35	−0.75	−1.08	−1.86
	RE/LHF	0.69	0.12	−0.03	0.83	0.06	0.92
	LE/RHF	0.17	0.57	0.27	−0.69	−0.46	−0.66
	LE/LHF	−0.39	−0.34	0.29	−0.03	0.11	0.41
B	RE/RHF	0.31	0.29	0.67	1.08	0.34	0.87
	RE/LHF	***2.63***	1.52	1.04	1.43	−0.18	0.55
	LE/RHF	1.53	1.14	0.77	1.12	***2.37***	***2.65***
	LE/LHF	1.43	0.68	1.48	0.22	0.54	0.41
C	RE/RHF	−1.94		0.93	−0.17		0.06
	RE/LHF	−0.04		0.51	−0.29		−0.65
	LE/RHF	−1.24		−***2.76***	−1.29		−1.28
	LE/LHF	−0.90		−0.99	−0.82		0.42
D	RE/RHF	−0.09		1.08	−0.71		−1.06
	RE/LHF	−1.66		**−*2.13***	−0.94		−1.08
	LE/RHF	−0.27		0.61	−0.85		−0.25
	LE/LHF	−1.64		−1.64	−1.01		−1.33

Rows contain z-scores for each condition with columns segregating the scores by cue location. Each condition is denoted by the viewing eye (RE, right eye; LE, left eye) and target location (RHF, right hemi-field; LHF, left hemi-field). Bolded and italicized z-scores indicate values greater than two standard deviations. The orientation of the cue location columns corresponds to the orientation of all panels in Figure 2.

**FIGURE 3:**
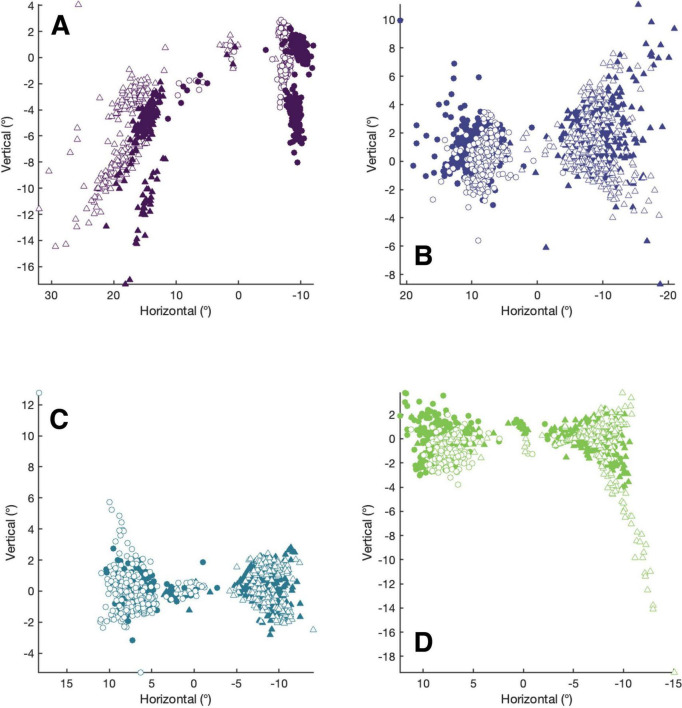
The accuracy of patients’ saccadic landing positions depends on viewing eye and target location. Each patient’s collection of saccadic landing positions, obtained from recording their sound eyes and shown in the equivalently named panels **(A–D)**, are plotted to differentiate the sound vs. affected eyes (filled vs. unfilled) and sound vs. affected hemi-field (circles vs. triangles) viewing conditions independent of cue location. The axis limits are set to the range of each patient’s data to enhance visualization.

We next considered that the memory-guided nature of the saccade task, designed to limit visual feedback and adaptation, influenced saccadic accuracy (Krappmann et al., 1998). The first set of analyses, which grouped normalized latencies by cue location alone, assumes that participants and patients executed saccades which landed at the visual target location. Since the spatial distance between saccadic landing positions and cue-locations best predicts latency modulations (Khan et al., 2010), our original grouping by cue locations alone could have obscured facilitatory or inhibitory effects if either participants or patients executed inaccurate movements. Figure 3, a scatterplot of each patient’s saccade landing positions, shows that the sound eye did typically execute accurate saccades toward visual targets. However, Patients A and D did make more inaccurate saccades when looking into their affected hemi-fields. Table 4, which contains the mean tangential errors for each patient, quantifies this observation. This is further expanded upon in Supplementary Figures 1–4 which show the trajectories of all saccades initiated by patients during Experiment I. These Supplementary figures first demonstrate that sound eye trajectories had spatiotemporal profiles similar to those of normal saccades, second that each patient’s fixation in each condition tended to be accurate and third, that, as in neurophysiological investigations, precedent visual cues have the potential to “curve” saccades away from subsequently presented targets. This phenomenon, observed when Patient D viewed with his sound right eye, is attributed to the competitive evolution of goal-selection in priority maps and is further evidence that paralytic strabismics contain neural circuits which dynamically integrate visuomotor information (McPeek et al., 2003). These findings motivated us to instead group participants’ and patients’ normalized latency distributions by the proximity of cues and saccade goals (i.e., landing positions). This was done by first computing the distance between saccade endpoints and cue positions for all conditions (the “cue-saccade offset”) and then sorting the normalized latency distributions into five respective bins: cue-saccade offsets between 0–5°, 5–10°, 10–15°, 15–20° and 20–25°.

**TABLE 4:** Tangential error for saccades.

Patient	Right hemi-field		Left hemi-field	
	Right eye	Left eye	Right eye	Left eye
A	9.75°	3.51°	8.24°	2.45°
B	2.48°	3.67°	2.53°	3.42°
C	4.12°	2.21°	2.97°	1.94°
D	4.84°	5.10°	4.21°	4.83°
*NS*	*2.82 – 3.21*°	*2.93 – 3.54*°	*2.63 – 3.42*°	*2.99 – 3.50*°

Rows contain the tangential error for each patient with columns segregating this data by target location (right or left hemi-field) and viewing eye (right eye or left eye) collapsed across cue positions. The ranges of mean tangential errors for non-strabismic (NS) participants, similarly collapsed across cue positions, are expressed as 95% confidence intervals in the last row.

**FIGURE 4:**
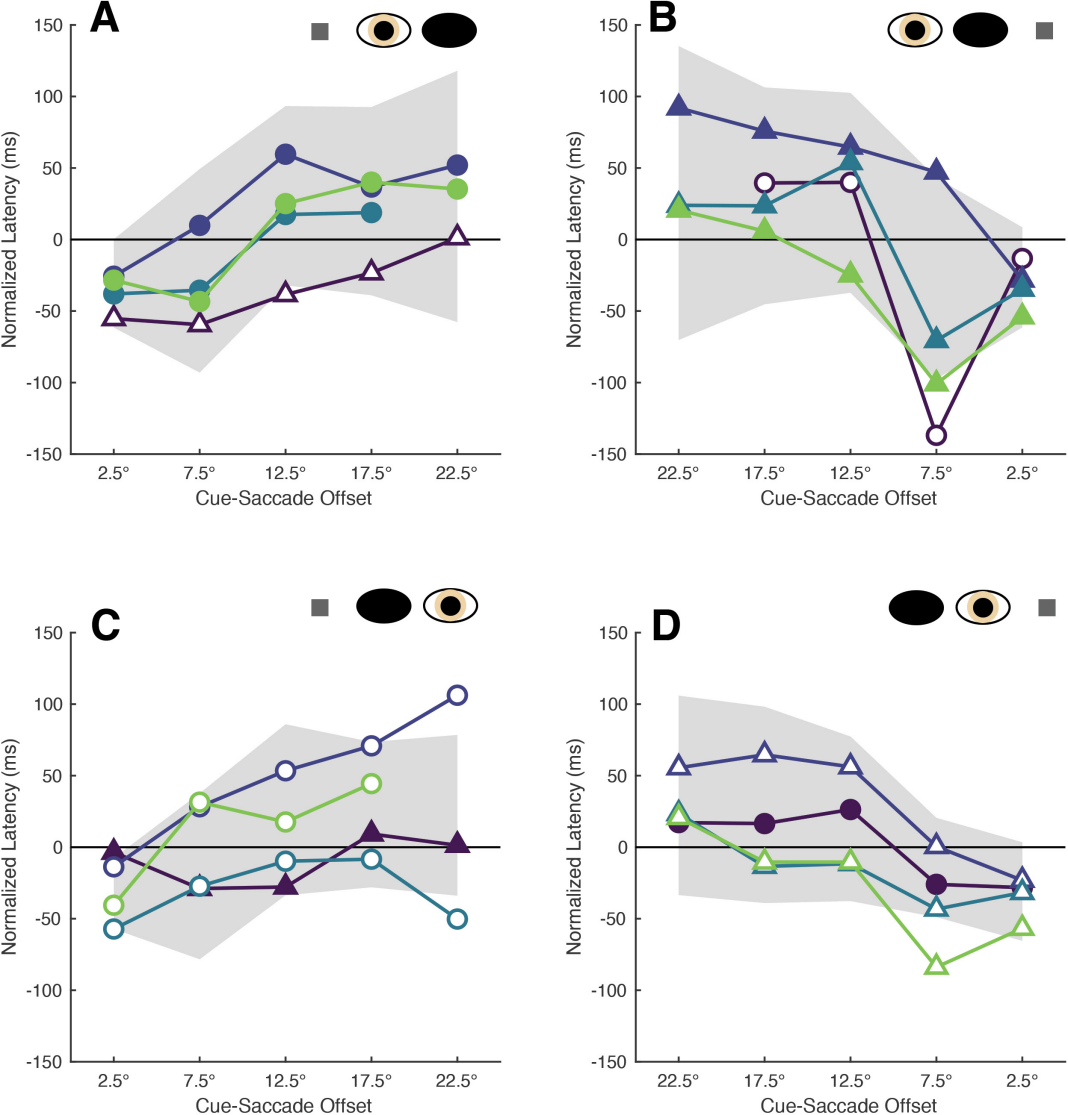
The latencies of patients’ saccades are modulated by cue-saccade offset. Each panel shows the mean normalized latencies of all patients (Patient A: purple, Patient B: dark blue, Patient C: blue, Patient D: green) for each viewing eye [right eye in panel **(A,B)**, left eye in panel **(C,D)**] and target location [rightward targets in panel **(A,C)**, leftward targets in panel **(B,D)**] combination. The graphing conventions match those used in Figure 2: panels are oriented such that one is viewing the patient, the shading and shape of datapoints differentiate the sound vs. affected eyes (filled vs. unfilled) and sound vs. affected hemi-fields (circles vs. triangles), and the confidence interval obtained from non-strabismic participants is shown in shaded gray. Labels on the abscissa indicate the midpoint of each respective cue-saccade offset bin. Datasets with four datapoints are those in which no saccades with offsets greater than 20° were executed.

Saccades planned closest to cue locations were executed fastest by both participants and patients (Figure 4). Kruskall-Wallis testing of the normative data confirmed a significant impact of cue-saccade offset on normalized latency: the tests revealed statistically significant differences in normalized latency across all viewing eye x target location conditions [H(4) = 23.68, 23.57, 27.07 and 22.77 for right eyes viewing right and left targets and left eyes viewing right and left targets with all *p* < 0.001, respectively]. *Post hoc* comparisons using the Tukey-Kramer method revealed that small cue-saccade offsets imparted significantly faster response latencies than larger cue-saccade offsets in our non-strabismic participants. This finding, summarized in Table 5, is supported by a similar “corner clustering” of significant results. This is to be expected given that the average saccadic error for non-strabismic individuals was approximately 3°. Therefore, because participants executed saccades which landed near the instructed targets, grouping by either cue location or cue-saccade offset produced similar results. Grouping patients’ normalized latencies by cue-saccade offset, which accounts for the periodic inaccuracy of saccade execution in this group, produced a pattern of results that closely matched those of the non-strabismic participants (Table 6): saccades directed closest to cues were initiated faster than those directed further away. The small number of patients’ datapoints which did fall greater than two standard deviations from the respective non-strabismic mean did not show a consistent direction of deviation. In summary, because most saccades were accurate, latency modulations observed with either grouping method reflect the integration and competition between visually-evoked cue activity and impending saccadic motor plans in both patients and participants.

**TABLE 5:** Results of *post-hoc* testing comparing non-strabismic participants’ mean normalized saccadic latencies across cue-saccade offsets within each viewing eye and target location condition.

Condition	*Post-hoc* comparisons					
RE/RHF		2.5	7.5°	12.5°	17.5°	22.5°
	2.5°			*	*	*
	7.5°			*		
	12.5°	*	*			
	17.5°	*				
	22.5°	*				
RE/LHF		2.5	7.5°	12.5°	17.5°	22.5°
	2.5°			*	*	*
	7.5°			*	*	*
	12.5°	*	*			
	17.5°	*	*			
	22.5°	*	*			
LE/RHF		2.5	7.5°	12.5°	17.5°	22.5°
	2.5°			*	*	*
	7.5°			*	*	
	12.5°	*	*			
	17.5°	*	*			
	22.5°	*				
LE/LHF		2.5	7.5°	12.5°	17.5°	22.5°
	2.5°			*	*	*
	7.5°					
	12.5°	*				
	17.5°	*				
	22.5°	*				

Asterisks indicate significant comparisons (*p* < 0.05) within each viewing eye (RE, right eye; LE, left eye) x target location (RHF, right hemi-field; LHF, left hemi-field) condition. Row and column labels indicate the mid-point of each cue-saccade offset bin.

**TABLE 6:** Z-scores for individual patients’ normalized saccadic latencies across cue-saccade offsets.

Patient	Condition	Cue-saccade offset				
		0–5°	5–10°	10–15°	15–20°	20–25°
A	RE/RHF	−1.60	−1.06	**−*2.21***	−1.52	−0.66
	RE/LHF	0.75	−***3.12***	0.21	0.24	
	LE/RHF	***2.33***	−0.30	−1.81	−0.53	−0.75
	LE/LHF	0.16	−0.68	0.22	−0.38	−0.55
B	RE/RHF	0.33	0.89	0.93	0.30	0.50
	RE/LHF	−0.11	***2.12***	0.92	1.19	1.16
	LE/RHF	1.52	1.68	0.91	1.89	***2.99***
	LE/LHF	0.45	0.84	1.26	1.02	0.55
C	RE/RHF	−0.47	−0.38	−0.42	−0.24	−0.17
	RE/LHF	−0.47	−1.24	−0.61	−0.18	
	LE/RHF	**−*2.01***	−0.24	−1.20	−1.23	**−*2.58***
	LE/LHF	−0.04	−1.68	−1.09	−1.25	−0.37
D	RE/RHF	0.15	−0.6	−0.18	0.4	0.12
	RE/LHF	−1.58	***−2.10***	−1.65	−0.65	−0.23
	LE/RHF	−0.66	−1.79	−0.29	0.85	−0.45
	LE/LHF	−1.49	***−4.02***	−1.05	−1.17	

Rows contain z-scores for each condition with columns segregating the scores by cue-saccade offset. Each condition is denoted by the viewing eye (RE, right eye; LE, left eye) and target location (RHF, right hemi-field; LHF, left hemi-field). Empty cells indicate conditions in which no saccades with cue-saccade offsets greater than 20° were executed. Bolded and italicized z-scores indicate values greater than two standard deviations.

### 3.2 Experiment II

The mean no-cue keypress latencies, expressed as 95% confidence intervals, ranged between 534 and 573 ms (right eye viewing right target), 570 to 593 ms (right eye viewing left target), 495 to 535 ms (left eye viewing right target) and 495 to 527 ms (left eye viewing left target) in non-strabismic individuals and were significantly different when compared across these conditions [H(3) = 12.13, *p* = 0.007 via Kruskall-Wallis test]. The keypress latencies initiated when non-strabismic individuals viewed targets in the left hemi-field with the right eye were significantly slower than all keypress latencies initiated in response to targets viewed with the left eye. Patients’ mean no-cue keypress latencies, reported in the same order, were as follows: Patient A (459, 463, 432 and 470 ms), Patient B (563, 588, 580 and 605 ms), Patient C (776, 700, 655 and 624 ms), and Patient E (620, 619, 605 and 634 ms).

**FIGURE 5:**
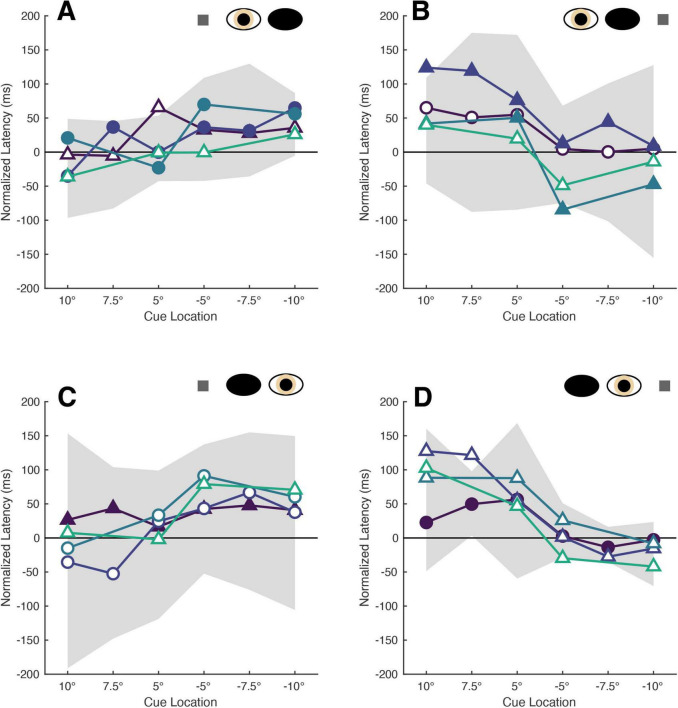
The latencies of patients’ keypresses are modulated by cue position. Each panel shows the mean normalized latencies of all patients (Patient A: purple, Patient B: dark blue, Patient C: blue, Patient E: teal) for each viewing eye [right eye in panel **(A,B)**, left eye in panel **(C,D)**] and target location [rightward targets in panel **(A,C)**, leftward targets in panel **(B,D)**] combination. Graphing conventions are equivalent to those used in Figures 2, 3: panels are oriented such that one is viewing the patient and the shading, shape of datapoints differentiate the sound vs. affected eyes (filled vs. unfilled) and sound vs. affected hemi-fields (circles vs. triangles) and the confidence interval obtained from non-strabismic participants is shown in shaded gray.

**FIGURE 6:**
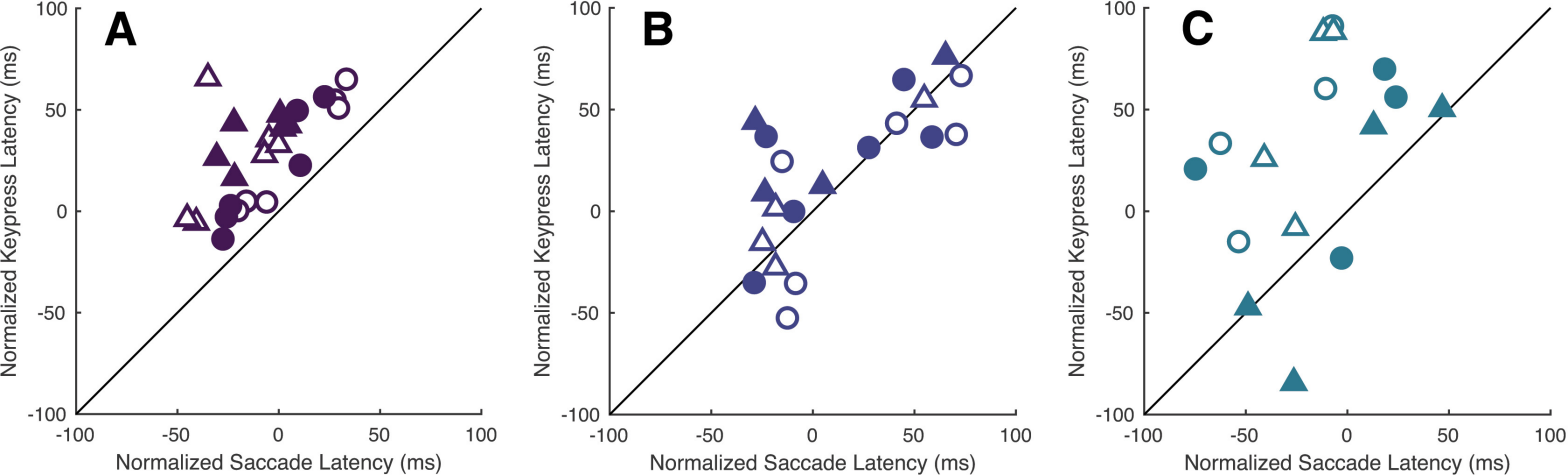
Cues modulate patients’ latencies similarly across response modalities. Panels **(A–C)** compare each respective patient’s normalized saccade and keypress latencies within each viewing-eye and target location condition. The shading and shape of the datapoints differentiate the sound vs. affected eyes (filled vs. unfilled) and sound vs. affected hemi-fields (circles vs. triangles).

We again accounted for potential hemi-field and/or viewing-eye dependent differences in baseline alertness through normalization of the cue-present latencies. This revealed a similar interaction between cue and target locations, mirroring the findings expressed in Experiment I, though keypress latencies were generally more variable in both participants and patients (Figure 5). Kruskall-Wallis tests conducted to examine differences in normalized keypress latencies across cue positions did reveal a significant influence of cue location in all viewing eye x target location conditions [H(5) = 28.44, 12.61, 17.02 and 37.42 for right eyes viewing right and left targets and left eyes viewing right and left targets with all *p* < 0.05, respectively]; however, *post-hoc* testing of this data garnered from non-strabismic individuals showed that the modulatory influence of cues was comparatively weaker than in the saccadic response modality. Significant comparisons were found but limited to the right eye/right target hemi-field and left-eye/left target hemi-field conditions (Table 7). Most patients exhibited idiosyncratic patterns of both facilitation and inhibition, with cues on the same side as targets generally reducing latencies, and cues on the opposite side increasing them, in a manner like that of non-strabismic individuals (Table 8). Crucially, as with saccades, cues closest to target locations consistently produced the greatest reductions in keypress response latencies while those furthest away consistently increased them, with patients’ rare deviations from the control group occurring in the direction predicted by the presence of exogenous spatial attention. The former result is illustrated by Figure 6 which compares the normalized saccade and keypress latencies of Patients A-C, who partook in both experiments. Spearman correlations revealed significant associations between normalized latencies in each response modality in two of the three patients (ρ = 0.65, 0.74 and 0.49 with *p* < 0.001 for Patients A & B and *p* = 0.056 for Patient C) showing that shorter normalized saccade latencies tended to be associated with shorter normalized keypress latencies and vice-versa. The comparatively smaller magnitude of facilitation in the keypress paradigm is demonstrated by the majority of each patient’s data falling above the reference lines.

**TABLE 7:** Results of *post-hoc* testing comparing non-strabismic participants’ mean normalized keypress latencies across cue locations within each viewing eye and target location condition.

Condition	*Post-hoc* comparisons						
RE/RHF		10°	7.5°	5°	−5°	−7.5°	−10°
	10°				*	*	*
	7.5°					*	*
	5°						
	−5°	*					
	−7.5°	*	*				
	−10°	*	*				
RE/LHF		10°	7.5°	5°	−5°	−7.5°	−10°
	10°						
	7.5°						
	5°						
	−5°						
	−7.5°						
	−10°						
LE/RHF		10°	7.5°	5°	−5°	−7.5°	−10°
	10°						
	7.5°						
	5°						
	−5°						
	−7.5°						
	−10°						
LE/LHF		10°	7.5°	5°	−5°	−7.5°	−10°
	10°					*	*
	7.5°					*	*
	5°					*	*
	−5°						
	−7.5°	*	*	*			
	−10°	*	*	*			

Asterisks indicate significant comparisons (*p* < 0.05) within each viewing eye (RE, right eye; LE; left eye) x target location (RHF, right hemi-field; LHF, left hem-field) condition.

**TABLE 8:** Z-scores for individual patients’ normalized keypress latencies across viewing eye and target location conditions.

Patient	Condition	Right cues			Left cues		
		10°	7.5°	5°	−5°	−7.5°	−10°
A	RE/RHF	0.56	0.41	***2.51***	−0.01	−0.46	−0.22
	RE/LHF	0.86	0.11	0.17	0.22	0.01	0.26
	LE/RHF	0.53	1.04	0.49	0.00	0.14	0.30
	LE/LHF	−0.63	−0.05	0.03	−0.41	−0.33	0.89
B	RE/RHF	−0.31	1.73	−0.23	0.08	−0.38	1.05
	RE/LHF	***2.38***	1.15	0.50	0.44	0.88	0.32
	LE/RHF	−0.19	−0.49	0.63	0.02	0.47	0.25
	LE/LHF	1.37	***3.01***	0.01	−0.48	−1.41	0.35
C	RE/RHF	1.23		−1.19	0.97		0.67
	RE/LHF	0.27		0.10	***−2.28***		−0.47
	LE/RHF	0.05		0.80	1.03		0.60
	LE/LHF	0.62		0.58	0.75		0.66
E	RE/RHF	0.34		−0.26	−0.90		−0.64
	RE/LHF	0.22		−0.38	−1.28		0.00
	LE/RHF	0.31		0.15	0.77		0.76
	LE/LHF	0.90		−0.14	***−2.04***		−0.79

Rows contain z-scores for each condition with columns segregating the scores by cue location. Each condition is denoted by the viewing eye (RE, right eye; LE, left eye) and target location (RHF, right hemi-field; LHF, left hemi-field). Bolded and italicized z-scores indicate values greater than two standard deviations. The orientation of the cue location columns corresponds to the orientation of all panels in Figure 5.

## 4 Discussion

Our patients’ data shows that exogenous spatial attention remains intact despite deficits in motor execution. Preceding visual cues modulated response latencies across most viewing eye and target hemi-field conditions and in both response paradigms. Notably, cues closest to target locations facilitated responses, while those furthest away induced inhibition. These effects may have been obscured in past studies which probed for cueing effects with a small number of cue and target locations. Further, given that recordings from patients’ sound eyes revealed generally accurate saccades, it is unlikely that a true absence of motor programming underlies the deficits previously described. Instead, our findings indicate that paralytic strabismics continue to plan movements when viewing with either eye and that dysfunction of a peripheral motor effector does not eliminate attention toward the effector’s region of motor space. Rather than questioning whether spatial attention operates in individuals with paralytic strabismus, we propose future research should instead investigate how. The framework of neural priority maps provides a promising scaffold for interpreting these findings, a concept which we explore next.

The comparable latency modulations observed in both response modalities, saccades and keypresses, suggest that patients integrated cue-evoked visual activity with both ocular and manual motor plans. This supports the idea that priority maps guiding sensorimotor integration operate independently of the specific effector used. If priority is allocated in effector-independent coordinates, then the presence of a paralysis may not impact the spatial location to which attention is directed but instead impact the kinematics of the recruited motor effectors (Zelinsky and Bisley, 2015). For example, in patients with sixth nerve palsies, after a goal-directed movement is selected via dynamic competition within the priority map, either the eyes and/or head may be utilized to direct a movement to that location in space. This would allow organisms to maintain circuits which dynamically update important spatial locations without regard for the underlying motor systems, and moreover, if overt movement is required, allow individual organisms to optimize the specific combination of effectors recruited for a given spatial location.

The individualized patterns of facilitation and inhibition observed across patients further support the idea that attention is shaped, not eliminated, by motor impairments. This points to a potential plasticity within the competitive circuitry of priority maps, allowing them to adapt to altered motor capabilities. These patient-specific adaptations could be formalized using mathematical models of priority maps (White et al., 2017), which may help identify the neural mechanisms which underlie person-specific patterns of reshaping. For example, whereas non-strabismic participants showed facilitation and inhibition from cues in discrete regions of space, Patient A’s saccades were facilitated by a broader spatial envelope, suggesting a lower resolution integration of visuomotor signals. This adaptation echoes the coarser sensory representations seen in amblyopia, where visual maldevelopment leads to degraded fine-scale processing. By analogy, the altered sensorimotor experiences of paralytic strabismics may contribute to the development of a similarly coarse-grained priority map. A related potential area for future investigation is whether the temporal dynamics of attention are similarly “blurred” in motor impaired individuals. Understanding both aspects of attentional allocation, where and when neural resources are deployed, and how they interact (i.e., speed-accuracy tradeoffs), is critical to grasping how individuals with motor impairments interact with their environment.

For decades, the attentional systems of those with muscle pareses have been assumed non-functional due to disrupted motor execution. Our findings challenge this view. They show that exogenous spatial attention is altered, but not absent, and highlight the need for future behavioral and computational investigations that account for neural plasticity in the face of ocular motor impairments. Theories which do not assume obligatory linkages between motor planning, execution and attentional allocation (Klein, 2020) are better suited to explore these questions than the strict hypotheses of the past. The framework of priority maps, distributed across both cortical and sub-cortical structures, is amenable to this flexibility because such structures display dynamic and competitive activity in spatially structured populations of visual, visuomotor and motor neurons. Therefore, we suggest that our data gathered from a small subset of paralytic strabismics should motivate neuroscientists and clinicians to first explore the spatio-attentional function of paralytic strabismics and other movement-restricted individuals across all regions of space before forming hypotheses based on their observed limitations.

## Data Availability

The raw data supporting the conclusions of this article will be made available by the authors, without undue reservation.

## Supplementary material

Supplementary Figure 1 Sound eye saccade trajectories for Patient A. Each panel shows the horizontal (solid lines) and vertical (dotted lines) components of all saccades analyzed in Experiment I. Sound eye trajectories are segregated by viewing eye [right eye in panel **(A,B)**, left eye in panel **(C,D)**] and target location [rightward targets in panel **(A,C)**, leftward targets in panel **(B,D)**] conditions as in previous figures. Positive values on the ordinate indicate rightward (horizontal) and upward (vertical) movements. The axis limits are set to the range of each patient’s data to enhance visualization.

Supplementary Figure 2 Sound eye saccade trajectories for Patient B. Each panel shows the horizontal (solid lines) and vertical (dotted lines) components of all saccades analyzed in Experiment I. Sound eye trajectories are segregated by viewing eye [right eye in panel **(A,B)**, left eye in panel **(C,D)**] and target location [rightward targets in panel **(A,C)**, leftward targets in panel **(B,D)**] conditions as in previous figures. Positive values on the ordinate indicate rightward (horizontal) and upward (vertical) movements. The axis limits are set to the range of each patient’s data to enhance visualization.

Supplementary Figure 3 Sound eye saccade trajectories for Patient C. Each panel shows the horizontal (solid lines) and vertical (dotted lines) components of all saccades analyzed in Experiment I. Sound eye trajectories are segregated by viewing eye [right eye in panel **(A,B)**, left eye in panel **(C)** and **(D)**] and target location [rightward targets in panel **(A,C)**, leftward targets in panel **(B,D)**] conditions as in previous figures. Positive values on the ordinate indicate rightward (horizontal) and upward (vertical) movements. The axis limits are set to the range of each patient’s data to enhance visualization.

Supplementary Figure 4 Sound eye saccade trajectories for Patient D. Each panel shows the horizontal (solid lines) and vertical (dotted lines) components of all saccades analyzed in Experiment I. Sound eye trajectories are segregated by viewing eye [right eye in panel **(A,B)**, left eye in panel **(C)** and **(D)**] and target location [rightward targets in panel **(A,C)**, leftward targets in panel **(B,D)**] conditions as in previous figures. Positive values on the ordinate indicate rightward (horizontal) and upward (vertical) movements. The axis limits are set to the range of each patient’s data to enhance visualization.
