# Supplementary figures and images for: Exogenous spatial attention is functional in paralytic strabismics
Source: Front Neurosci. 2025 Sep 22;19:1650468. doi: 10.3389/fnins.2025.1650468 (PMC12497859; doi:10.3389/fnins.2025.1650468)

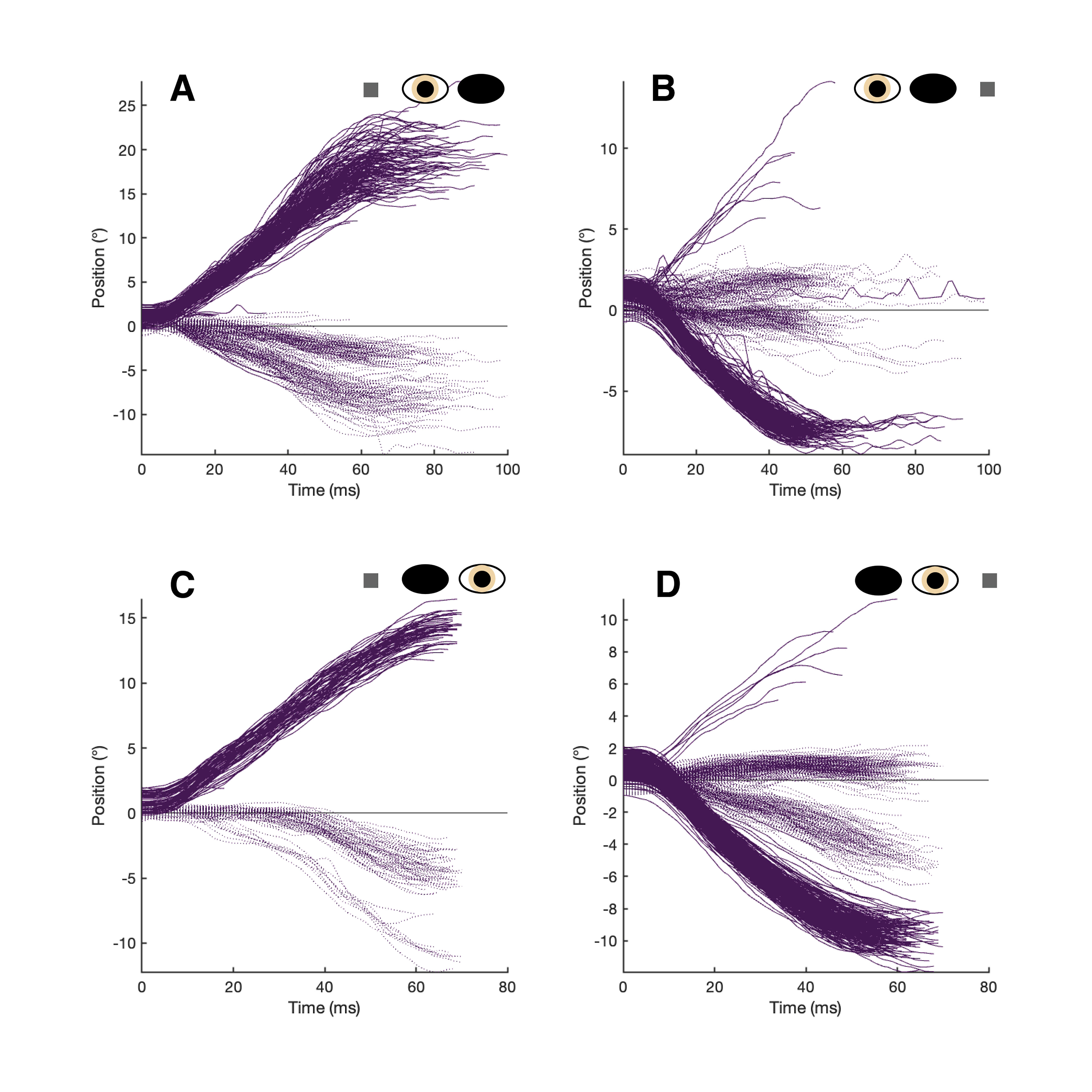

Supplement: Supplementary Figure 1 — Sound eye saccade trajectories for Patient A. Each panel shows the horizontal (solid lines) and vertical (dotted lines) components of all saccades analyzed in Experiment I. Sound eye trajectories are segregated by viewing eye [right eye in panel (A,B), left eye in panel (C,D)] and target location [rightward targets in panel (A,C), leftward targets in panel (B,D)] conditions as in previous figures. Positive values on the ordinate indicate rightward (horizontal) and upward (vertical) movements. The axis limits are set to the range of each patient’s data to enhance visualization. [file Image_1.jpeg]

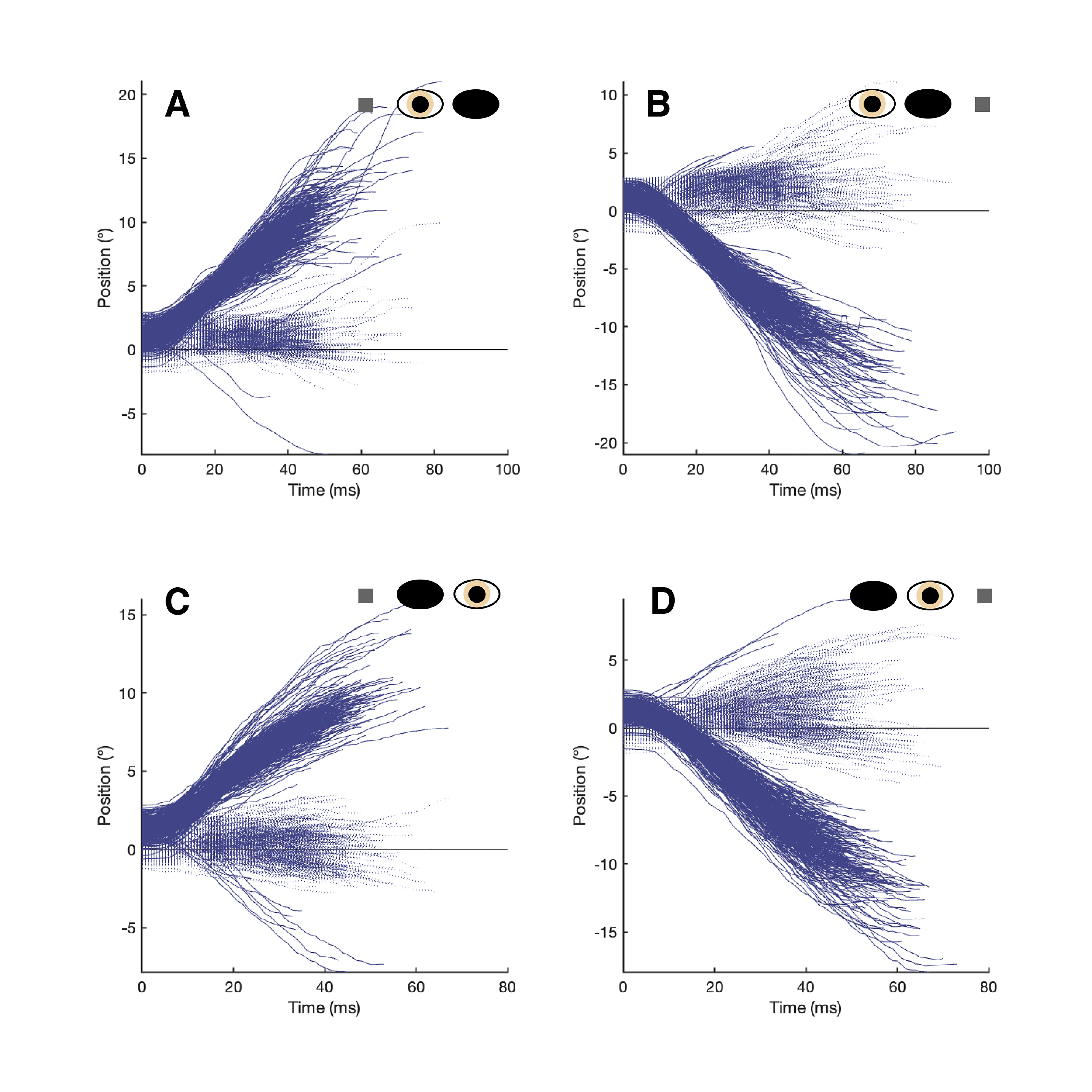

Supplement: Supplementary Figure 2 — Sound eye saccade trajectories for Patient B. Each panel shows the horizontal (solid lines) and vertical (dotted lines) components of all saccades analyzed in Experiment I. Sound eye trajectories are segregated by viewing eye [right eye in panel (A,B), left eye in panel (C,D)] and target location [rightward targets in panel (A,C), leftward targets in panel (B,D)] conditions as in previous figures. Positive values on the ordinate indicate rightward (horizontal) and upward (vertical) movements. The axis limits are set to the range of each patient’s data to enhance visualization. [file Image_2.jpeg]

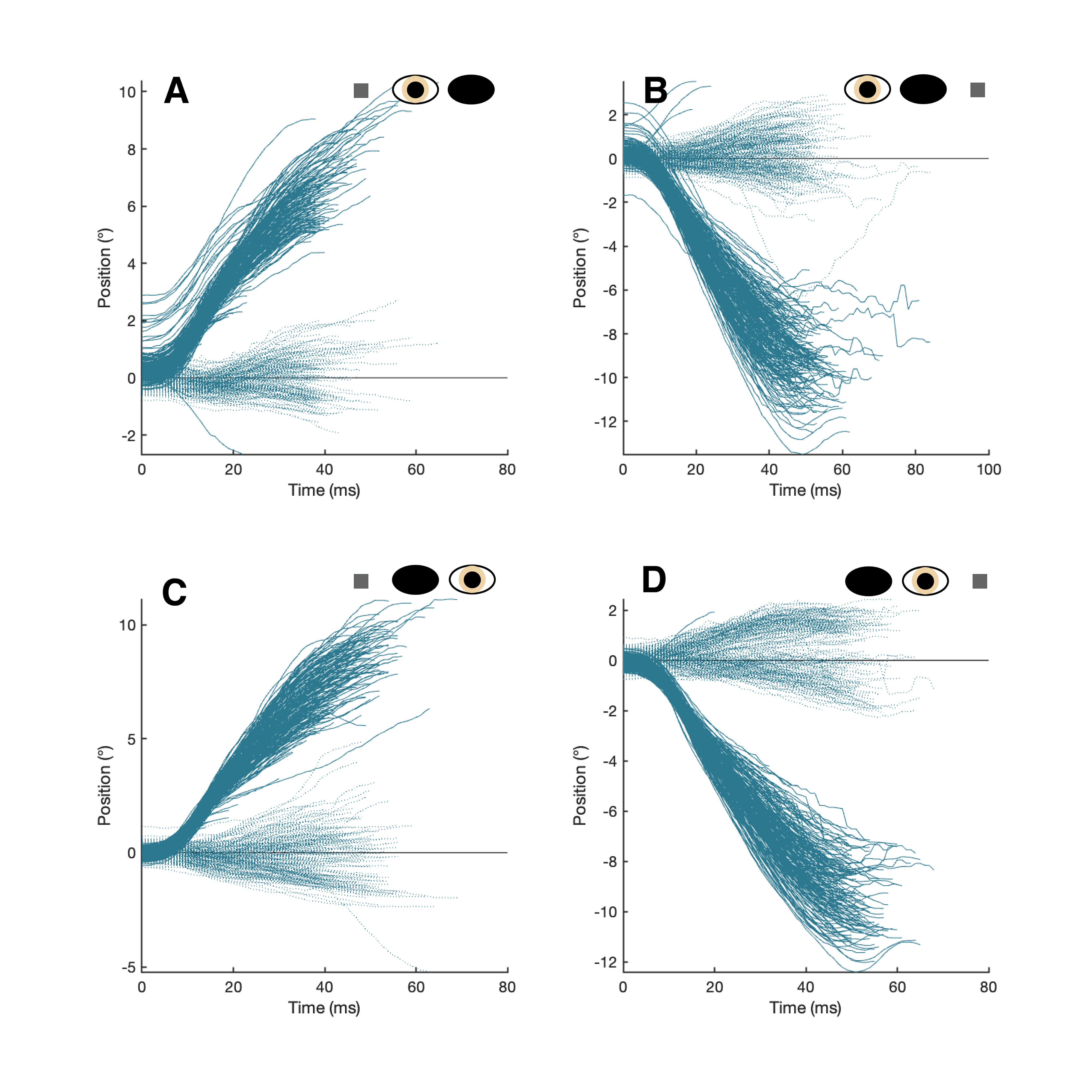

Supplement: Supplementary Figure 3 — Sound eye saccade trajectories for Patient C. Each panel shows the horizontal (solid lines) and vertical (dotted lines) components of all saccades analyzed in Experiment I. Sound eye trajectories are segregated by viewing eye [right eye in panel (A,B), left eye in panel (C) and (D)] and target location [rightward targets in panel (A,C), leftward targets in panel (B,D)] conditions as in previous figures. Positive values on the ordinate indicate rightward (horizontal) and upward (vertical) movements. The axis limits are set to the range of each patient’s data to enhance visualization. [file Image_3.jpeg]

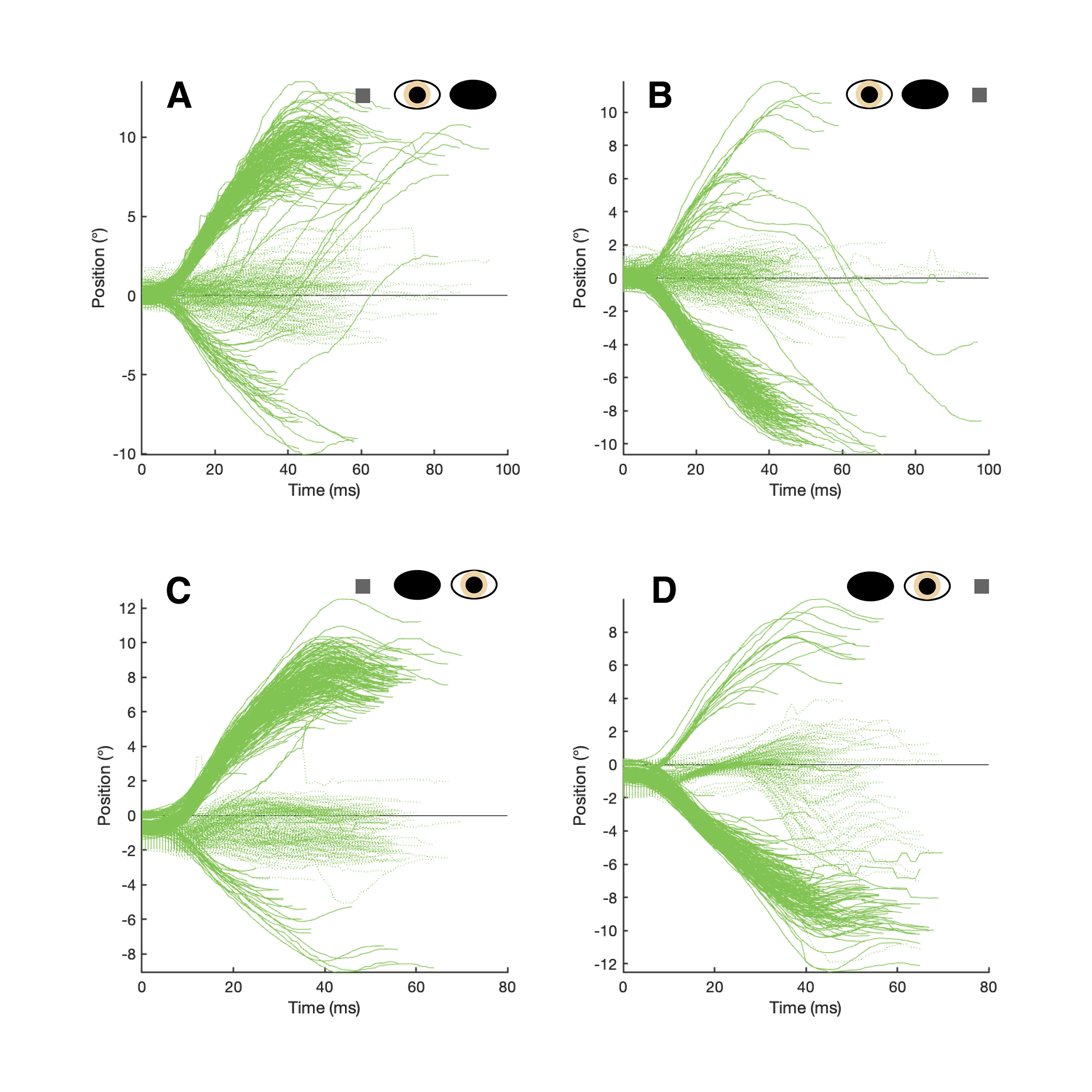

Supplement: Supplementary Figure 4 — Sound eye saccade trajectories for Patient D. Each panel shows the horizontal (solid lines) and vertical (dotted lines) components of all saccades analyzed in Experiment I. Sound eye trajectories are segregated by viewing eye [right eye in panel (A,B), left eye in panel (C) and (D)] and target location [rightward targets in panel (A,C), leftward targets in panel (B,D)] conditions as in previous figures. Positive values on the ordinate indicate rightward (horizontal) and upward (vertical) movements. The axis limits are set to the range of each patient’s data to enhance visualization. [file Image_4.jpeg]
